# Development of a novel morphological paclitaxel-loaded PLGA microspheres for effective cancer therapy: *in vitro* and *in vivo* evaluations

**DOI:** 10.1080/10717544.2017.1422296

**Published:** 2018-01-04

**Authors:** Zongrui Zhang, Xinyu Wang, Binbin Li, Yuanjing Hou, Jing Yang, Li Yi

**Affiliations:** ^a^ State Key Laboratory of Advanced Technology for Materials Synthesis and Processing, Wuhan University of Technology Wuhan China; ^b^ Biomedical Materials and Engineering Research Center of Hubei Province, Wuhan University of Technology Wuhan China; ^c^ School of Foreign Languages, Wuhan University of Technology Wuhan China; ^d^ Institute of Textiles and Clothing, The Hong Kong Polytechnic University Hung Hom, Kowloon, Hong Kong P.R. China

**Keywords:** Paclitaxel, morphological features, sustained release, antitumor efficacy, cancer therapy

## Abstract

Sustained release of therapeutic agents into tumor cells is a potential approach to improve therapeutic efficacy, decrease side effects, and the drug administration frequency. Herein, we used the modified double-emulsion solvent evaporation (DSE) method to prepare a novel morphological paclitaxel (PTX) loaded poly(lactide-co-glycolide) (PLGA) microspheres (MS). The prepared rough PTX-PLGA-MS possessed microporous surface and highly porous internal structures, which significantly influenced the drug entrapment and release behaviors. The rough MS with an average particle size of 53.47 ± 2.87 μm achieved high drug loading (15.63%) and encapsulation efficiency (92.82%), and provided a favorable sustained drug release. The *in vitro* antitumor tests of flow cytometry and fluoroimmunoassay revealed that the rough PTX-PLGA-MS displayed effective anti-gliomas activity and enhanced the cellular PTX uptake through adsorptive endocytosis. Both *in vitro* and *in vivo* antitumor results demonstrated that the sustained-release PTX could induce the microtubules assembly and the over-expression of Bax and Cyclin B1 proteins, resulting in the microtubule dynamics disruption, G2/M phase arrest, and cell apoptosis accordingly. Furthermore, as the rough PTX-PLGA-MS could disperse and adhere throughout the tumor sites and cause extensive tumor cell apoptosis with one therapeutic course (12 days), they could reduce the system toxicity and drug administration frequency, thus achieving significant tumor inhibitory effects with rapid sustained drug release. In conclusion, our results verified that the rough PTX-PLGA-MS drug release system could serve as a promising treatment to malignant glioma.

## Introduction

Glioma is the most aggressive and frequent common intracranial malignant tumor, comprising approximately 50% of all intracranial tumors and causing cancer-related death worldwide (Yang & Wang, 2016; Wang et al., 2017). Glioma developed from multipotent cancer stem cells has the tendency of over-proliferation and possesses the capacity to proliferate into heterogeneous lineages of cancer cells under specific stimuli (Xie et al., 2014). Currently, the common approaches for treating glioma patients are surgical resection (Pessina et al., 2016), localization irradiation (Navarria et al., 2016), and targeted chemotherapy (Murai et al., 2016). However, the successful treatments are still remarkably limited by the conventional chemotherapy resistance and the frequency of drug use (Guo et al., 2011; Floyd et al., 2016). Therefore, the development of novel strategies to achieve effective chemotherapy becomes essential in the treatment of malignant glioma.

Paclitaxel (PTX) as a new class of microtubule stabilizing agent, has shown significant antitumor efficacy against various tumors, including refractory ovarian, breast, lung, and other cancer types (Hou et al., 2017; Yu et al., 2017). PTX functions through the promotion of microtubules assembly and stabilization, thereby inhibiting mitosis and inducing cell apoptosis (Naraharisetti et al., 2007). However, its successful clinical cancer therapy is compromised by its poor aqueous solubility, drug resistance in tumor cells, and nonspecific pharmacokinetics in systemic circulation (Duan et al., 2017). Furthermore, the effective anti-gliomas efficacy of PTX is limited by its poor permeability across the blood-brain barrier (BBB) and the inability to maintain a higher drug concentration at the tumor sites (Zhan et al., 2010).

In an attempt to achieve high BBB penetration and glioma targeting abilities, researchers have developed the nanoparticles drug delivery system (DDS) with functional surface groups (Nikanjam et al., 2007). However, the complexity of these drug delivery strategies is increased, the high drug-loading content and targeted drug release cannot be achieved simultaneously. Interestingly, upon brain tumor treatments, different research groups have achieved superior antitumor efficacy by implanting drug-loaded MS directly into the precise and functional brain areas using stereotaxy (Jollivet, 2004; Menei et al., 2004). Since these studies, the development of polymeric PTX delivery implants with high drug-loading efficiency and sustained drug release is important existing in choosing suitable delivery vehicle (Joshi et al., 2016). Poly(lactide-co-glycolide) (PLGA) with excellent biocompatibility and adjustable degradation periods could be ideal as delivery vehicle and the highly hydrophobic PTX could be readily incorporated into hydrophobic PLGA matrices (Ho et al., 2008).

Nowadays, most publications focus on the drug-loading capacity and release behaviors of drug-loaded microspheres (MS) better than their morphology which is strongly correlated to drug entrapment and release behaviors (Bile et al., 2015; Obayemi et al., 2016). To research how the MS morphology exerts effects, researchers have prepared porous drug-loaded MS with hollow core-shell structure and smooth MS with denser matrix structure (Bae et al., 2010). The smooth MS with narrow hydrophobic space resulted in less PTX packaging and the drug release hindered by the nonporous surface leaded to drug resistance increase (Shiny et al., 2013). While, by comparison, the hollow core-shell MS achieved higher drug loading quantity because of enough hydrophobic space for PTX-loading (Wang et al., 2015). Additionally, these highly porous MS displayed an initial burst and a much faster drug release diffusing from the mesoporous MS matrices, whereas the smooth MS showed slow drug release due to the lower inner porosity and longer diffusion lengths (Bae et al., 2010; Lee et al., 2010).

In most cases, the burst drug release was attributed to the MS surface drugs dispersion or the porous drug diffusion. The sustained drug delivery possessed the advantage of prolonging drug release time, protecting drugs from biological activity loss, and reducing administration frequency accordingly (Kokai et al., 2010; Feng et al., 2014). The fundamental understanding of the drug release mechanisms is essential to prepare the novel morphological rough PTX-PLGA-MS, because the drug release kinetics dominantly depend on the morphology and drug distribution of MS (Yang et al., 2001). As delivery carriers of PTX, their morphology has significant influences on drug entrapment and release behaviors, which further affects antitumor activity (Wang et al., 2015). Furthermore, the disruption of programed cell proliferation signaling pathways may result in elevated cell death, so the changes of molecule biology in glioma cells play important roles in malignant glioma treatment (Mercurio et al., 2017).

**Figure 1.**
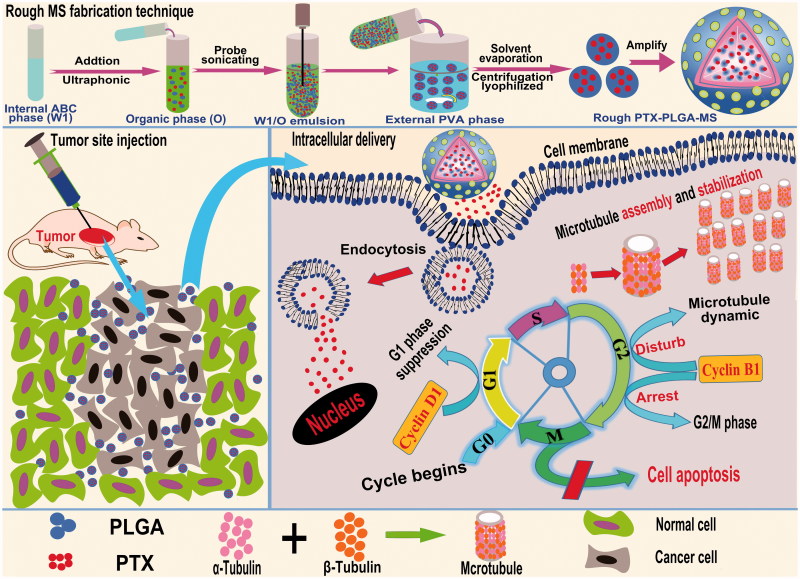
Schematic representation of the rough PTX-PLGA-MS fabrication technique, tumor site injection and intracellular drug delivery pathway. Intracellular trafficking includes enhanced PTX uptake through adsorptive endocytosis, sustained PTX release, and promoted microtubule assembly and stabilization. The dysregulation of cell cycle progression includes arresting the G2/M cell phase and disturbing microtubules dynamic equilibrium.

In this study, the special rough PTX-PLGA-MS were prepared by the modified double-emulsion solvent evaporation (DSE) technique and their morphology, drug entrapment and release behaviors were evaluated. These tests were carried out to confirm whether the rough MS possessed a desired morphological features, higher drug loading capacity, and enhanced sustained drug release activity. Additionally, the molecular mechanisms of rough MS against tumor cells (Figure 1) including cellular uptake of PTX, drugs induced cell apoptosis, and microtubules assembly had been elucidated using the flow cytometry, fluoroimmunoassay, and Western blot analysis. The *in vivo* tumor inhibition ratios were evaluated by directly injecting PTX formulations to the mice xenograft tumor sites and analyzing the volumes, weights, histology, and molecule biology of the treated tumors. The implications of these results above are thoroughly analyzed for the development of implantable rough PTX-PLGA-MS for optimal therapeutic efficacy inside solid tumors.

## Materials and methods

### Materials

PLGA (lactide/glycolide =75/25, molecular weight(M_w_) = 24,483) were synthesized in our laboratory. Paclitaxel (98% purity), polyvinyl alcohol (PVA, 89% hydrolyzed, M_w_ = 77,000), ammonium bicarbonate (ABC), dichloromethane (DCM), deuterated chloroform (CDCl_3_), trimethylsilane (TMS), phosphate-buffered saline (PBS), and sodium salicylate were purchased from Huashun Biological Technology Co., Ltd (Wuhan, China). Trypsin, penicillin, streptomycin, Dulbecco’s minimal essential medium (DMEM), Cell Counting Kit (CCK-8), paraformaldehyde, Triton X-100, annexin V/fluorescein isothiocyanate (FITC), propidium iodide (PI), 4,-diamidino-2-phenylindole (DAPI), and polyvinylidene difluoride (PVDF) membranes were all bought from Sigma, St. Louis, MO. Primary antibodies against glyceraldehyde 3-phosphate dehydrogenase (GAPDH), Bax, Bcl-2, Cyclin B1, and Cyclin D1 were purchased from Beyotime Biotechnology, Jiangsu, China. Horseradish peroxidase (HRP)-conjugated goat anti-mouse and anti-rabbit IgG secondary antibodies were also obtained from Sigma.

Human glioma (U251) and hepatoma (HepG2) cells were grown in DMEM medium supplemented with 10% (v/v) fetal bovine serum and antibiotic supplements (penicillin and streptomycin at both 100 units/mL), and was incubated in a humidified atmosphere (37 °C, 5% CO_2_). Female BALB/c-nu mice (14–16 g) were obtained from the Animal Center of Wuhan University. The mice were housed at 50% relative humidity (25 °C) for 12 h light/dark cycle and had free access to standard chow food. All animal experiments were performed in compliance with the guidelines set by the national regulations and approved by the ethical committee for animal care of the medical sector.

### Methods

#### Preparation of PTX-loaded PLGA microspheres

The rough PTX-PLGA-MS were prepared using double emulsion method (Figure 1) based on solvent evaporation as previously described with modification (Fang et al., 2014). Firstly, 10 mg of ABC were dissolved in 2 mL deionized water (DI) to prepare the internal water phase (W1). PTX (20 mg) and PLGA (200 mg) were initially dissolved in 6 mL DCM (oil phase, O). Then, the W1/O homogeneous emulsion was formed by ultrasonic dispersing in an ice-water bath for 60 s. After probe sonicating, the resultant emulsion was transferred into 100 mL 2 wt% PVA solution (external water phase, W2) under magnetic stirring and was continuously stirred at 800 rpm to remove the solvent. The solidified MS were centrifuged (2000 rpm) and washed four times with DI to remove PVA and non-incorporated drugs. Afterwards, the washed MS were lyophilized and stored in a vacuum desiccator at −20 °C for further analysis. The smooth PTX-PLGA-MS were prepared by the O/W2 emulsion method with PVA concentration at 0.5 wt%. Additionally, the blank PLGA-MS were prepared by the same parameters without addition of PTX drugs.

##### ^1 ^H NMR and morphology analysis

^1 ^H NMR spectra were recorded on a Bruker AVANCE 500MHZ (AV 500, Germany) spectrometer at 400 MHz (25 °C), taking CDCl_3_ as eluent and TMS as internal reference. For morphological studies, MS were sprinkled on a double-sided adhesive tape previously applied to an aluminum stub and then fixed onto a graphite surface. The samples were coated with a 30 nm layer of gold and visualized under scanning electron microscope(SEM; Zeiss Ultra Plus, Germany) at 25 kV. Synchrotron radiation X-ray microtomography scans (SR-μCT, Shanghai Synchrotron Radiation Facility (SSRF), BL13W1) were conducted to investigate the internal and microstructure of these MS. The MS were blended in a micropipette container and scanned with SR-μCT at 15 keV. Pixel size was 3.25 μm, sample-to-detector distance was 12 cm, and exposure time was 5 s. The total projected images were reconstructed using X-TRACT SSRF CWS × 64. Moreover, the three dimension (3 D) rendered data were analyzed with VGStudio Max (Version 2.1, Volume Graphics GmbH, Germany) and Image Pro Analyzer 3 D (Version 7.0, Media Cybernetics, Rockville, MD).

#### *Particle size, drug-loading capacity, and* in vitro *release experiments*

The mean diameter and polydispersity index of drug-loaded MS were measured using a Malvern Instrument (Mastersizer 3000, England) based on laser light scattering. Particle sizes and size distributions were determined by examining a homogeneous MS ultra-purified water suspension and were confirmed by measuring the MS diameters on their SEM micrographs using ImageJ software. Their drug loading (DL) and encapsulate efficiency (EE) were analyzed using an ultraviolet spectrometer (UV-2550, SHIMADZU, Japan). Firstly, the calibration curve of PTX was determined by detecting a range of standard PTX concentrations (0–100 μg/mL). The high correlation coefficient (*R* ^2^ = 0.99958) fitted by Origin software confirmed the satisfied standard curve. Then, 10 mg MS were dissolved in 5 mL DCM with stirring overnight to ensure thorough dissolution. 10 mL methanol was added into the above solution, and the mixture was vortexed for 1 min before being measured by UV detection. The actual DL and EE were calculated using Equations (1) and (2):

(1) DL (%)=(Weight of drugs in MS) / (Weight of MS) × 100%

(2) EE (%)=Actual DL(%) / Theoretical DL(%) × 100%

For release experiments, 15 mg PTX-loaded MS were suspended in 5 mL sodium salicylate/PBS (pH 7.4) medium (1 mol/L) in a dialysis membrane (M_w_ = 14,000 Da). For free PTX release behavior, 2 mg PTX were suspended in the medium. The dialysis bags were separately immersed into a screw-capped tube with 50 mL of the aforementioned solution, which then was placed horizontally in an orbital shaker (HZQ-F160, Harbin, China). At predetermined time intervals, 2 mL of the solutions in the tube was collected for UV absorbance analysis at 227 nm. Thereafter, the tubes were replenished with 2 mL fresh medium and placed back inside the shaker. The drug release amount was estimated through monitoring the absorbance using the above calibration Equations (1) and (2). Additionally, morphological examinations of these MS after drugs release for 3, 7, and 14 days were undertaken using SEM.

#### In vitro *cytotoxicity and antitumor activity*

The delivery vehicle cytotoxicity was evaluated by seeding U-251 human glioblastoma cells in 96-well plates (3 × 10^3^ cells/well) in 200 μL DMEM. For antitumor tests, the cell density was set as 5 × 10^3^ cells/well, and 20 μL fresh PBS was employed as control group. After 24 h incubation, PTX formulations with equivalent PTX concentrations (0.01–100 μg/mL in 20 μL PBS) were added to each well. After 48 h treatment, the 96-well plates were replenished with 200 μL DMEM and 10 μL CCK-8 solution, and then incubated in a 5% CO_2_ atmosphere (37 °C) for another 2 h. The solution absorbency was measured on an ELISA (Multiskan, Thermo Fisher, Finland) micro-plate reader at 450 nm. Cell viability was calculated using Equation (3):

(3) $$Cell\;viability\;(\%)=(A_{sample}-\;A_{PBS})\;/\;(A_{PBS}-\;A_{cell})\;\times \;100\%$$

Where *A*_sample,_ *A*_PBS,_ and *A*_cell_ represent the absorbencies of the sample, the PBS medium and the cells, respectively. According to the results, the values of the drug concentration at inhibition of 50% cell growth (IC_50_) were calculated using SPSS(Chicago, IL, version 17.0).

#### Cell cycle and apoptosis analysis

The cell cycle and apoptosis induced by the treatments of free PTX and rough PTX-PLGA-MS were evaluated by fluorescence-activated cell sorter (FACS) analysis (BD, Biosciences, Franklin Lakes, NJ). Briefly, U251 cells (100 × 10^4^ cells/mL) were seeded in a culture flask (25 cm^2^) and was treated with samples for 48 h. Thereafter, cells were harvested by trypsin digestion, washed twice with cold PBS (4 °C), centrifuged (1500 rpm) for 5 min, and fixed in ethanol solution (70% v/v, 4 °C) for at least 24 h. The fixed cells were collected and resuspended in 500 μL PBS containing Triton-X 100 (0.3% v/v), annexin V-FITC (200 mg/L), and PI (15 mg/L). After incubation in the dark at 37 °C for 30 min, cell apoptosis and cell cycle phase changes was analyzed using the FACS equipped with the Cell Quest software.

#### Fluoroimmunoassay

For the immunofluorescence assay, free PTX, and rough PTX-PLGA-MS with PTX concentration of 5 μg/mL were incubated in six-well plates which were previous seeded with U251 cells (5 × 10^4^ cells/well). After 24, 48, and 72 h of incubation, cells were fixed in paraformaldehyde (4% v/v, 30 min), permeabilized with Triton-X 100 (0.3% v/v, 10 min), and blocked in normal goat serum (5% v/v, 30 min). Primary incubation was conducted with a collagen primary antibody (in 3% BSA, 1:500, α-tubulin mouse antihuman) at 4 °C overnight, followed by 90 min secondary antibody incubation (in 3% BSA, 1:500, α-tubulin-FITC goat antimouse, 37 °C lucifuge). Cell nuclei were stained with DAPI (10 μg/mL) for 10 min away from light. The images of stained cells were observed with a fluorescence microscope (IX71, OLYMPUS, Tokyo, Japan) and the NIS-Elements software.

#### Western blot analysis

Western blot analysis on U251 cells were subjected to different treatments: control group, free PTX, and rough and smooth PTX-PLGA-MS. After 24 and 48 h treatment, the protein lysates were harvested by grinding the treated cells in ice-cold PBS solution. The homogenate were dissolved in radio immunoprecipitation assay (RIPA) buffer and centrifuged (12,000 rpm) for 10 min (4 °C). Protein concentration was quantitated by the BCA protein assay kit and equal amounts (30 μg) of cellular proteins were separated by 10% (v/v) sodium dodecyl sulfate polyacrylamide gel electrophoresis (SDS-PAGE). The GAPDH antibody was served as the internal loading control. Proteins were transferred to a PVDF membrane, which then was blocked with 5% (v/v) nonfat dry milk in tris-buffered saline and Tween 20 (TBST) for 4 h (37 °C) and incubated with a specific primary antibody overnight (4 °C). The blots were washed thrice in TBST buffer and incubated with an appropriate HRP-labeled secondary antibody for 2 h (4 °C). Specific protein detections were achieved using the BCIP (5-bromo-4-chloro-3-indolyl-phosphate) / NBT (nitro blue tetrazolium) Alkaline Phosphatase Color Development Kit according to the manufacturer instructions. The intensity bands of proteins were quantified by the Quantity One software.

#### In vivo *antitumor activity*

To determine whether the rough PTX-PLGA-MS obtained improved *in vivo* therapeutic efficacy, the tumor xenograft models were created by subcutaneously inoculating freshly harvested HepG2(human liver carcinoma) cells in the right flank region of BALB/c-nu mice (3 × 10^6^ cells/mice) to allow for easy and reproducible tumor volume measurements. Tumor volumes were measured by calipers and calculated as *V* =* d* ^2^ × *D*/2 (*d* and *D* represent the shortest and the longest tumor diameter, respectively). When tumor volumes reached a mean size of approximately 260 mm^3^, the mice were randomly divided into five groups and were administered via intratumoral injection. As the cumulative PTX release amount of rough MS was 85% for 12 days, the rough and smooth MS were injected every 12 days, while the free PTX was injected every four days. In order to compare the free PTX and drug-loaded MS, different injections but the same whole dose should be chosen. The free PTX treatment dosage was set as 10 mg/kg and the dosage of drug-loaded MS were chosen at 30 mg PTX equiv./kg accordingly. Additionally, the control groups of saline and empty PLGA-MS were administered every four days. After treatments, the tumor volumes and body weights of all tumor-bearing mice were recorded every four days. At the end of the experiment, the excised tumors were photographed and weighted. The tumor growth rate was calculated as tumor growth volumes/days (mm^3^/day). For tumor histology, the collected cryosection tumor tissues (10 μm in thickness) were stained with hematoxylin and eosin (HE) and examined under a fluorescence microscope equipped with green filter.

#### In vivo *molecular biology studies*

Quantitative real-time polymerase chain reaction (qRT-PCR) was used for the assessment of mRNA expression of the aforementioned apoptosis and cell cycle genes. Total RNA of the treated tumor tissue was extracted using the RNeasy Kit (Qiagen, Hilden, Germany). The reverse transcription reaction (RTR) was performed using Superscript III (Invitrogen, Carlsbad, CA) in a final volume of 20 mL containing 5 mg of the total RNA. After 50 min incubation (37 °C), the RTR was terminated by heating at 72 °C for 15 min. The newly synthesized cDNA was amplified using the PCR synthesis kit (Invitrogen) and the PCR fragments were visualized by ethidium bromide staining. Changes in the threshold method were used to calculate the relative mRNA expression and the results were normalized to the endogenous control GAPDH for gene expression analysis. The primer sequences are displayed in Supplementary Table S1. Protein expression levels of different treatments on liver tumors were evaluated by Western blot. Firstly, tumor tissues after treatment were homogenized, dissolved in RIPA buffer, and centrifuged (12,000 rpm) for 10 min (4 °C). The following test steps were according to the *in vitro* Western blot method.

#### Statistical analysis

All numerical data were given as the mean ± standard deviation. At least three replicates were set up in each group and two independent experiments were performed. The results were statistically analyzed using one-way analysis of variance (ANOVA) through SPSS. *p* values less than .05 were considered statistically significant.

## Results

### PTX-PLGA-MS preparation and characterization

**Figure 2.**
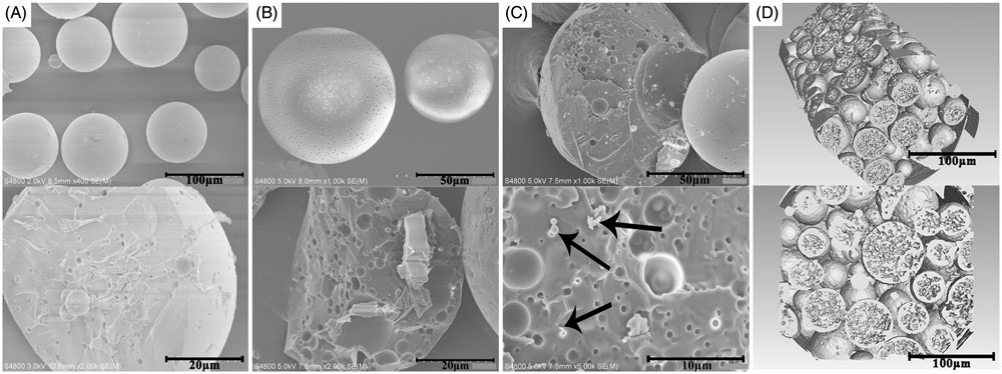
The surface and internal SEM micrographs of (A) smooth PTX-PLGA-MS and (B) rough PTX-PLGA-MS; (C) The fractured internal morphology of rough PTX-PLGA-MS. The arrows represent the PTX drug substances; and (D): The 3 D outer and inner morphometric of rough PTX-PLGA-MS.

In this study, a novel morphological rough PTX-PLGA-MS were prepared using the modified DSE method. Generally, the double emulsion is an unstable system and the solvent diffusion evaporation from W1/O emulsion to W2 aqueous phase has significant impacts on the surface and inner morphology of MS (Fang et al., 2014). The ^1 ^H NMR spectra (Supplementary Figure S1) were obtained to identify their characteristic functional groups. The signals observed at 1.59, 4.85, and 5.18 ppm are referred to protons of methyl, methylene, and methyne groups of PLGA polymers correspondingly. Meanwhile, signals arising from methyl (1.27 ppm), acetyl (1.71–2.41 ppm), and aromatic (7.38–8.16 ppm) protons are observed in the spectrum of free PTX. Importantly, all the signals of polymer and drugs are visible in drug-loaded MS spectrum. These observations indicated that PTX was successfully encapsulated into PLGA-MS. Figure 2(A) showed that the smooth PTX-PLGA-MS were spherical in shape with internal sporadic porosity. While, the rough MS (Figure 2(B)) exhibited microporous surface and porous internal structures. The PTX substances observed in the fractured internal morphology of rough MS (Figure 2(C)) also confirmed the successful drug entrapment. Furthermore, the 3 D cross-section images indicated that the rough MS were porous internal matrices with big holes (Figure 2(D)). The rough PTX-PLGA-MS prepared with a higher external PVA concentration showed a homogeneous internal porous structure and micropores surface with deep surface folds compared to that of smooth MS.

### *Particle size, drug-loading capacity, and* in vitro *release analysis*

An ideal drug carrier should have a narrow size distribution, high DL, and EE (Jeffery et al., 1993). In this study, as the process parameters (the aqueous phase volume, agitation speed, PLGA M_w_, composition, and concentration) were the same, the main difference was the use of high external PVA concentration in rough MS preparation. As a result, the rough MS achieved smaller particle sizes (approximately 50 μm) and narrower size distributions than that of smooth MS (Supplementary Table S2). Generally, the uniformly distributed MS particle sizes had slight effects on the DL and EE of MS. Moreover, the novel morphological rough PTX-PLGA-MS achieved higher drug-loading efficiency (15.63%) and encapsulation efficiency (92.82%) than the smooth MS (D.L = 10.85% and E.E = 85.01%). According to the morphology analysis results, unlike smooth MS with internal sporadic porosity, the rough MS with highly porous internal structure could provide enough hydrophobic space for PTX packaging.

**Figure 3.**
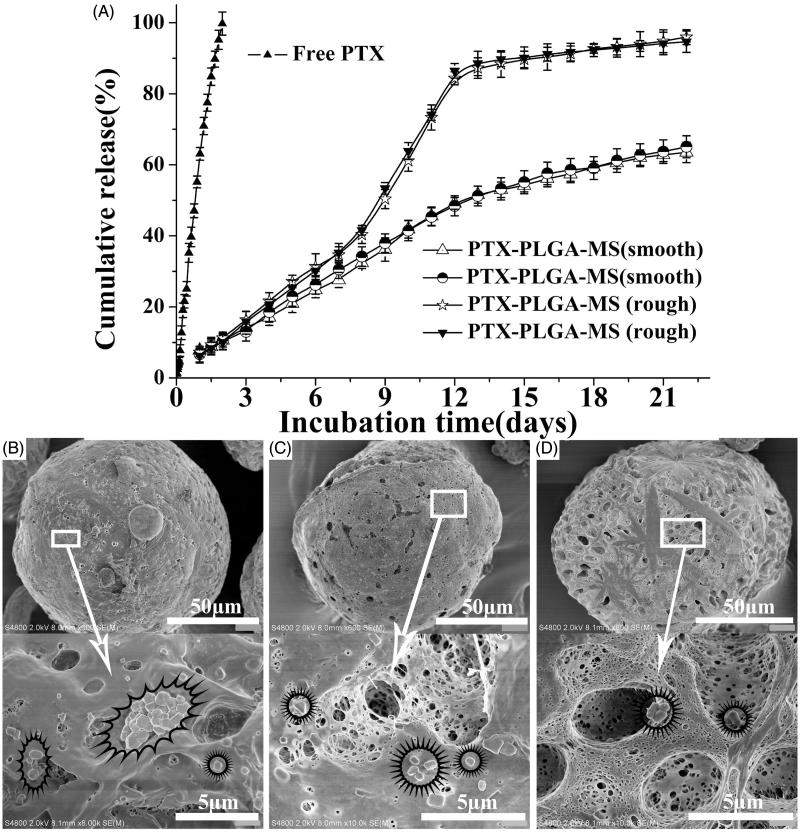
(A) *In vitro* cumulative released curves of free PTX, smooth, and rough PTX-PLGA-MS. SEM micrographs of rough PTX-PLGA-MS after incubation in the release medium for (B) three, (C) seven, and (D) 14 days. The stars mark the PTX crystals on the mesopores of MS.

The release of free PTX (Figure 3(A)) was observed at an zero-order release phase and the drug release amount was about 85% at 48 h. Whereas, the rough PTX-PLGA-MS exhibited S-curve release patterns and the cumulative PTX release amount was approximately 11 and 85% at 48 h and 12 days, respectively. The smooth MS release patterns were found to be approximately linear with correlation coefficients from 0.962 to 0.965. Additionally, the release rate of smooth MS was slower than that of rough MS, which might be attributed to the slow matrices degradation rate and internal sporadic porosity of smooth MS. The SEM micrographs of drug released smooth MS after seven days incubation (Supplementary Figure S2) showed that the MS are still intact without hollow bores and the hollow bores were not found until released for 14 and 21 days, thus confirming their slow matrices degradation rate. Noteworthy, the rough MS showed a fast drug release for seven days and leveled off at day 14. After being released for seven days, the rough MS particle sizes increased and the spherical MS became deformed with considerable mesopores (Figure 3(C,D)). The rough MS appeared increasingly porous and aggregated with longer immersion times. The results above indicated that the rough MS with deep surface folds achieved a faster drug release rate than the smooth MS.

### In vitro *antitumor activity*

The cytotoxicity results showed that the polymer displayed negligible cytotoxicity and the cells incubated with empty PLGA-MS even at the highest concentration exhibited normal growth state (Supplementary Figure S3). Whereas, the PTX formulations displayed cytotoxic potency to the proliferation of U251 cells in a dose-dependent manner. Interestingly, when U251 cells were incubated with smooth and rough PTX-PLGA-MS at equivalent PTX concentrations, the cell viability of smooth MS were lower than that of rough MS group. The smooth MS treated group showed a high IC_50_ at 9.73 ± 0.42 μg/mL, indicating their lower potent antitumor activity. While the rough MS treated group exhibited a lower IC_50_ (4.15 ± 0.38 μg/mL) than free PTX group (5.60 ± 0.35 μg/mL), which suggested that the rough MS with sustained drug release achieved enhanced antitumor effects rather than a sharp inhibition caused by free PTX.

### Cell cycle and apoptosis analysis

**Figure 4.**
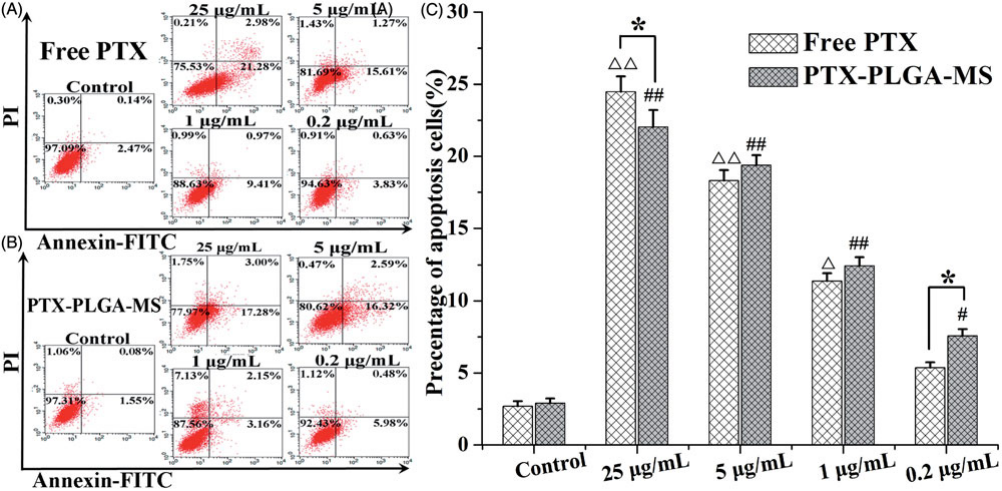
Apoptosis analysis of U251 cells induced by (A) free PTX and (B) rough PTX-PLGA-MS with different concentrations of PTX (25, 5, 1, and 0.2 μg/mL, respectively) for 48 h using flow cytometry. (C) The bar graphs show the percentage of cell apoptosis with different treatments. △, # vs. control group, △, *,#*p* < .05, and △△, ##*p* < .02.

The U251 cells apoptosis and cycle changes after incubation with PTX formulations for 48 h were evaluated using flow cytometry assay. As shown in Figure 4(A,B), the cell apoptosis percentage treated by the highest concentration of free PTX and rough PTX-PLGA-MS were 24.47 ± 1.07 and 22.03 ± 1.16%, respectively. Interestingly, with a lower PTX concentration, the rough MS caused higher cell apoptosis than free PTX (Figure 4(C)), suggesting their effective drug delivery on promoting cell apoptosis. Supplementary Table S3 showed that the percentage of cells in the G2/M phase treated by PTX drugs significantly increased and a small portion of cells were arrested in the S phase. Importantly, a low-dose rough MS displayed a large proportion of cells arrested in the G2/M phase. The further increase in PTX concentration resulted in a great decrease in the viable number of cells, only a few viable cells were in the G0/G1 phase.

### Fluoroimmunoassay and Western blot analysis

The immunofluorescence assay were conducted to examine the intracellular drug accumulation and subsequent drug distribution in U251 cells. The blue and green channel represented the DAPI-stained nuclei and the drug induced microtubules expression in the cytoplasm, respectively (Supplementary Figure S4). The cells induced by free PTX and rough PTX-PLGA-MS exhibited typical apoptotic features, such as bright staining, nuclei fragmenting, and chromatin condensing nuclei (Lin et al., 2013). Furthermore, the rough MS treated group showed a prolongation of microtubules expression for the sustained drug release. In order to research how the multifunctional proteins contribute to the tumor inhibition in glioma, the immunoblotting were performed. The cells in free PTX and rough MS treated groups showed a higher expression of Bax and Cyclin B1, and a lower expression of Bcl-2 (Supplementary Figure S5) than that of control group. In smooth MS group, all the protein expressions displayed a negligible variation, whereas the Bax and Cyclin B1 expressions in rough MS group apparently increased (Supplementary Figure S5 (C,D)). Additionally, the proteins expression levels were displayed in a time-dependent manner except for the Cyclin D1 protein levels.

### In vivo *antitumor efficacy*

**Figure 5.**
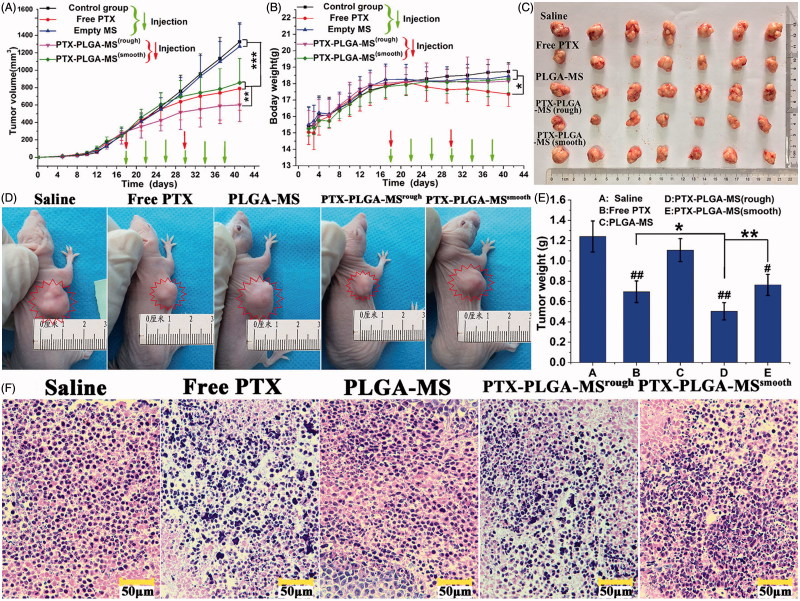
*In vivo* antitumor efficacies of saline, free PTX, empty PLGA-MS, rough, and smooth PTX-PLGA-MS in tumor-bearing nude mice (*n* = 7). (A) Tumor growth curves during the entire experiment. (B) Body weight variations of mice. Photographs of (C) excised tumors and (D) tumor-bearing nude mice at the end of the treatment procedure. (E) Weights of excised tumor at the end of the tests. (F) The histological characteristics of liver tumor tissue after treatments. # vs. control group, *, # *p* < .05; **, ##*p* < .02; and ***p < .01.

The treatment dosage was calculated as free PTX dosage at 10 mg/kg, which was between the mean arterial blood pressure and maximum tolerated dose (Zhong et al., 2016). Figure 5(A) displayed the tumor volume changes for saline and empty PLGA-MS treated mice, which indicated they both had no therapeutic effect. However, the tumor volumes of PTX formulations groups slightly increased, and the rough MS group (602.65 ± 193.75 mm^3^) showed the smallest final tumor volume. Noteworthy, mice treated with free PTX exhibited noticeable body weight loss and hypersensitivity symptoms (Figure 5(B)). While, the body weight of MS-treated groups slightly increased and became stable at around 18.1 g. The photographs (Figure 5(C,D)) of excised and mice-bearing liver tumors showed a direct intuitive proof of the remarkable tumor inhibition effect of rough MS. Additionally, the final tumor weight for rough MS group was smaller than that of free PTX and smooth MS groups (Figure 5(E)). Tumor inhibition efficiency was calculated based on the tumor weight and volume measurements, the rough PTX-PLGA-MS inhibited tumor growth efficiently (59.27%), which was 1.35- and 1.55-fold higher than that of free PTX and smooth MS, respectively. The HE staining images (Figure 5(F)) showed that the tumors treated by PTX preparations were composed of poorly cohesive heterogeneous cells, which were mostly polyhedral in shape, and had a large nucleus/cytoplasm index. However, the control groups exhibited noticeably higher tumor cell density, negligible necrosis, and sporadic cell apoptosis.

### In vivo *molecular biology studies*

**Figure 6.**
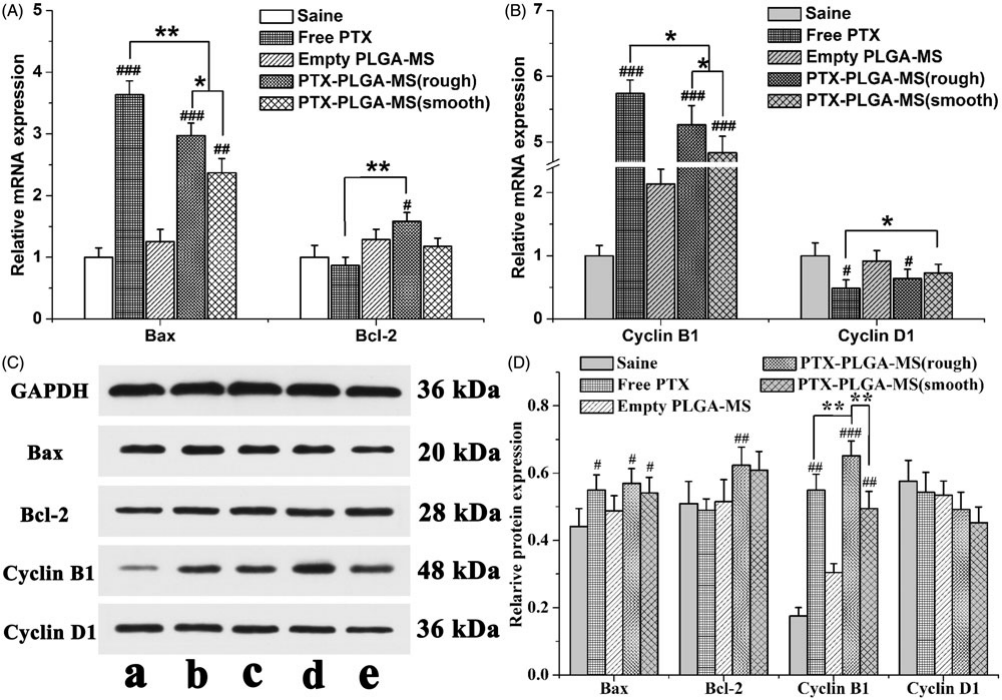
Molecular biological analysis of liver tumors receiving different treatments. Relative (A) Bax/Bcl-2 and (B) Cyclin B1/Cyclin D1 mRNA expression changes in liver tumors were analyzed by PCR. GAPDH was used as an internal reference. (C) Effects of saline, free PTX, empty PLGA-MS,) rough PTX-PLGA-MS, and smooth PTX-PLGA-MS on proteins expression in liver tumors. (D) Quantitative evaluation on proteins expression were further analyzed by densitometry. # vs. saline group; *, #*p* < .05; **, ##*p* < .02; and ###*p* < .01.

Subsequent to the tumor growth inhibition analysis, the molecular biology studies were performed to evaluate the gene expressions of cell cycle and pro-apoptotic molecule pathways influenced by PTX drugs. Figure 6(A,B) showed that the PTX formulations groups exhibited higher Bax and Cyclin B1 gene expressions than control group. Additionally, the mRNA expressions level in rough PTX-PLGA-MS group was higher than that of smooth MS group. In line with the RT-PCR results, treatment of tumor tissues with PTX drugs have similar effects on Bax and Cyclin B1 proteins expression. These groups exhibited no obvious transcription changes of Bcl-2 and Cyclin D1 expression both at the mRNA and protein levels. Noteworthy, compare to free PTX group, the rough MS group exhibited higher Bax and Cyclin B1 proteins expression (Figure 6(D)). These results were accordant with the *in vitro* cell cycle and Western blot results, which indicated that the PTX-mediated mitotic arrest or cell cycle distributions were significantly affected by the activation of these proteins.

## Discussion

The favorable sustained drug release and high drug accumulation in tumor cells are the critical factors of DDS for achieving a superior antitumor efficiency. The current study developed rough PTX-PLGA-MS with microporous surface and internal porous structures to achieve these advantages. The ^1 ^H NMR spectra analysis demonstrated the successful drug encapsulation. The characteristic PTX peaks in drug-loaded MS were less prominent than that of free PTX, which might be because the PTX is trapped in porous internal core and isolated by PLGA matrices shell. The two key factors existing in the formation mechanism of the novel morphology are the use of ABC solution and higher external PVA concentration. As the high PVA concentration may stabilize the interfacial inherent thermodynamics between the two immiscible (W1/O and W2) phases, the spontaneous coalescence of the multi-core globules can be avoided by the collision between these two metastable emulsion droplets (Fang et al., 2014). Additionally, within a number of small W1/O droplets, the ABC solution tends to produce carbon dioxide, which is able to pass through the membranes between inner holes, thus producing internal porous morphology throughout the PLGA matrices. As the gases may further escape from the MS surface, leaving pores on the surface, the small W1/O droplets were encapsulated in the high viscosity PVA globule which may slow down the carbon dioxide diffusion rate, thus the prepared rough MS were endowed with deep surface folds. The microporous surface was attributed to the diffusion of DCM from the organic to the external PVA phases, which caused precipitation of the dissolved PTX at PLGA matrices during particle solidification. Unlike porous MS with hollow sphere pores (Kokai et al., 2010; Wang et al., 2015), these micropores were not interconnecting holes and might hinder the access and diffusion of release medium into MS, thus achieving low burst release. The specific aim of this work was to evaluate whether the unique morphology have significant influences on the PTX-loading quantity and *in vitro* PTX release behaviors.

Generally, the obtained MS particle size is mainly dependent on the size and stability of emulsion droplets formed during the stirring process. The small emulsion coagulation easily induced by specific rotational speed and shear forces became stable once formed and caused the MS to have a narrower size distribution. When setting the same stirring speed, a decrease in MS size can be achieved by increasing the external PVA concentration. The presence of high external PVA concentration could break up the emulsion into smaller droplets and stabilize emulsion droplets against coalescence by providing a hydrophilic environment during emulsification and solidification, resulting in smaller particle sizes and uniformed drug distribution within MS. Additionally, the hydrophobic PLGA matrices possessed a readily incorporation of hydrophobic PTX drugs. The rough PTX-PLGA-MS achieved higher DL and EE than the prepared smooth MS and other PTX-loaded MS prepared by former researchers (Du et al., 2006; Wang et al., 2016), because their internal porous structure could provide enough hydrophobic space for PTX-loading and the high PVA concentration could prevent the drugs from diffusing out. The sustained release of drugs from a carrier system is an important property for their biomedical application in cancer treatment. The rough PTX-PLGA-MS showed a faster drug release rate than those smooth MS, which might be because the rough MS with deep surface folds achieved a higher matrices degradation rate and the drug diffusion-migration from rough MS was accelerated by the immersion of the release medium into the matrices through the hollow bores. As the polymer matrices degrade, the release rate became fast due to the hollow bores diffusion of drugs from MS matrices. The effective drug release could be facilitated by the accelerated drug release rate, thus reducing the risk of drug resistance. Therefore, the rough PTX-PLGA-MS possessed the enhancement of drug activity, the relatively fast drug release, and the favorable sustained drug release.

*In vitro* antitumor results showed that the smooth MS showed higher antitumor efficacy only after 48 h incubation. This phenomenon may stem from the fact that the initial drug release for smooth MS was mainly attributed to the release of drugs distributed on the MS surface. While for the release of rough MS, the crystallized drug were released by simple dissolution and diffusion. As we know, the terminal half-life of PTX was about 5.8 h, so the obvious cell apoptosis observed in free PTX and smooth MS treated groups was indicative of the limitations of systemic drug administration because they only provided short and acute drug exposure to tumor cells. Noteworthy, the rough PTX-PLGA-MS showed higher antitumor efficacy than free PTX and smooth MS with prolongation of incubation, which suggested that the rough MS possessed the efficient transport of sustained-release PTX into tumor cells. As apoptosis deregulation is the hallmark of all cancer cells, the induction of apoptosis has been described as the standard, and best strategy in anticancer therapy (Strasser et al., 2011). The apoptosis results were in line with the *in vitro* antitumor activity, indicating that with lower PTX concentration, the rough PTX-PLGA-MS induced more early and late apoptosis. The growth inhibitory effect was also caused by a specific perturbation of cell cycle-related events. The cell cycle results suggested that the treatment of PTX formulations could disrupt cell division process and lead to cell apoptosis by arresting cells in the G2/M phase. The increase in G2/M phase arrest was attributed to the PTX caused microtubules assembly and stabilization, and the mitotic spindle function interference. The fact that the rough MS show continued inhibition to U251 cells rather than a sharp inhibition caused by free PTX demonstrated that they achieved the strengthened antitumor efficiency and the lower cytotoxicity to normal cells.

The drug uptake and diffusion throughout the tumor cells play a critical role in the antitumor effect of DDS. The green fluorescence channel completely overlaid the blue channel, indicating that the PTX drugs readily penetrated through the cells lipid membranes into the nuclei, thus showing diffuse cytoplasmic and nuclear fluorescence. This result suggested that the rough MS possessed the remarkable capability of drug uptake and transport into cancer cells through adsorptive endocytosis. Furthermore, the antitumor efficacy of rough MS could be facilitated by the expression of cell cycle and apoptosis related proteins. The Bax and Bcl-2 protein expression results coincided with the cell apoptosis data indicated that the rough MS exhibited prolonged proteins expression and inhibition effects. The Cyclin D1 expression levels were consistent with the cell cycle results that a few viable cells were in the G0/G1 phase. The high Cyclin B1 expression levels could induce increased chemosensitivity of U251 cells to PTX and also a delicate balance existed between nonspecific cell death and specific transfection efficiency. This balance is due partly to the Cdk1/Cyclin B1 mediated phosphorylation and inactivation of anti-apoptotic Bcl-2 protein. Therefore, the activation of pro-apoptotic signaling and the stabilization of microtubules are thought to be the principal mechanisms for the cytotoxic action of PTX drugs in inhibiting motility and invasion of U251 cells. The presented strategy of rough PTX-PLGA-MS system could mediate cell cycle proteins expression, thus receding the drug resistance in tumor cells and increasing the efficiency of chemotherapy.

Most importantly, PTX formulations on tumor inhibition were evaluated in liver tumor-bearing mice to see whether they could enhance the *in vivo* antitumor efficacy and reduce system toxicity. It is noteworthy that the rough PTX-PLGA-MS group (13.45 mm^3^/day) showed higher tumor growth inhibition potency than free PTX (21.92 mm^3^/day) and smooth MS (25.46 mm^3^/day) groups. This was because the rough MS possessed higher drug-loading capacity and rapid sustained drug release than the smooth MS. The noticeable body weight loss in free PTX group was in good agreement with prior reports (Ding et al., 2014; Yi et al., 2015), which was attributed to the high drug level in the blood and the nonselective drug uptake by normal tissues (Che et al., 2015). While, the stable body weight in MS-treated groups suggested their negligible side effects and were suitable for systemic chemotherapy. The necrosis areas (pyknosis, karyorrhexis) in HE images exhibited an extensive lymphocyte infiltration into the tumor tissue and a low presence of connective tissue (Perez et al., 2015). The free PTX group showed a negative antitumor effect because of the frequency of drug use caused high plasma drug concentration in the body. The smooth MS with lower drug-loading capacity and slower drug release rate resulted in insufficient drug accumulation in the tumor sites, thus achieving unsatisfactory antitumor efficiency. As the rough PTX-PLGA-MS could evenly disperse and adhere throughout the tumor sites, they caused the most extensive cell apoptosis and necrosis with one therapeutic course (12 days), thus reducing drug distribution in normal tissues, and achieving a significant inhibitory effect with rapid sustained drug release.

The molecular biology analysis clearly confirmed the improvement in antitumor efficiency of rough PTX-PLGA-MS. The observed increase expression in pro-apoptosis levels *in vivo* proved that the rough MS induced significant cell apoptosis. Additionally, the decreased cell proliferation rate observed in the Cyclin B1 overexpressing cells were consistent with the *in vitro* Western blot results, suggesting that Cyclin B1 protein level may affect cell cycle progression. The involvement of these signaling events verified that the expression of cell cycle and pro-apoptotic molecule pathways is essential for the enhancement of PTX toxicity in tumor tissues. Unlike the smooth MS group with slow drug release rate, the rapid sustained PTX release from rough MS enabled the tumor cells to sequential exposure to PTX drugs. The sustained accumulation of PTX drugs at the tumor sites will continuous kill the tumor cells by pro-expressing those cell cycle and apoptosis mRNA and proteins. The rough MS with highly chemotherapy sensitizing effect could increase drug accumulation and uptake in both inherent and acquired liver tumor cells. Based on these results, we concluded that the adsorptive endocytosis mediated drug delivery of rough PTX-PLGA-MS produced fewer drug resistance, thus achieving higher antitumor effects and less damage to normal tissues.

## Conclusion

To sum up, we developed the implantable rough PTX-PLGA-MS with microporous surface and porous internal structure to overcome recognized problems with PTX drug administration, such as the unfavorable drug release, undesirable drug accumulation, and drug resistance to tumors. Due to the unique morphology, the resultant rough MS achieved high drug-loading capacity and rapid sustained drug release. Moreover, the rough MS enhanced the cellular uptake and intracellular delivery of PTX, and actively induced the expressions of cell cycle and pro-apoptotic genes and proteins, thereby inhibiting cell proliferation, as well as increasing cell apoptosis. Importantly, the rough PTX-PLGA-MS not only exhibited effectively *in vitro* antitumor activity, but also significantly suppressed tumor growth, and improved animal survival by decreasing the drug administration frequency and side effects. Therefore, this rough PTX-PLGA-MS DDS represents a promising strategy to enhance the efficacy of malignant glioma therapy.

## Supplementary Material

IDRD_Wang_et_al_Supplemental_Content.docx
