# Supplementary material for: Development of a novel morphological paclitaxel-loaded PLGA microspheres for effective cancer therapy: in vitro and in vivo evaluations
Source: Drug Deliv. 2018 Jan 4;25(1):166–77. doi: 10.1080/10717544.2017.1422296 (PMC6058517; doi:10.1080/10717544.2017.1422296)
Supplement: IDRD_Wang_et_al_Supplemental_Content.docx [file IDRD_A_1422296_SM0332.docx]

**Development of a novel morphological paclitaxel-loaded PLGA microspheres for effective cancer therapy: *In vitro* and *in vivo* evaluations**

Zongrui Zhang^a,b^, Xinyu Wang^a,b,^*, Binbin Li^a,b^, Yuanjing Hou^a,b^, Jing Yang ^c^, Yi Li^d^

a State Key Laboratory of Advanced Technology for Materials Synthesis and Processing, Wuhan University of Technology, Wuhan 430070, China

b Biomedical Materials and Engineering Research Center of Hubei Province, Wuhan University of Technology, Wuhan 430070, China

c School of Foreign Languages, Wuhan University of Technology, Wuhan 430070, China

d Institute of Textiles and Clothing, The Hong Kong Polytechnic University, Hung Hom, Kowloon, Hong Kong, P.R. China

^#^These authors contributed equally to this manuscript.

Corresponding authors:

Xinyu Wang (Tel.: +86-13807128122, Fax: +86-027-87880734, E-mail addresses: wangxinyu@whut.edu.cn).

# ***In vivo molecular biology studies***

Table S1. The primer pairs of GAPDH, Bax, Bcl-2, Cyclin B1 and Cyclin D1 for real-time quantitative RT-PCR.

| Gene name | Forward primer | Reverse primer | Band size (bp) |
| --- | --- | --- | --- |
| GAPDH | AGGAGCGAGACCCCACTAACA | AGGGGGGCTAAGCAGTTGGT | 247 |
| Bax | GCCTTTTTGCTACAGGGTTTCAT | TATTGCTGTCCAGTTCATCTCCA | 151 |
| Bcl-2 | TGACTTCTCTCGTCGCTACCGT | CCTGAAGAGTTCCTCCACCACC | 112 |
| Cyclin B1 | GAACCAGAGGTGGAACTTGCT | CACTACAGAGGTTTGGGTCAGC | 229 |
| Cyclin D1 | ATCTCCTCAACGACCGGGTG | CGGCAGTCAAGGGAATGGT | 303 |

# PTX-PLGA-MS preparation and characterization


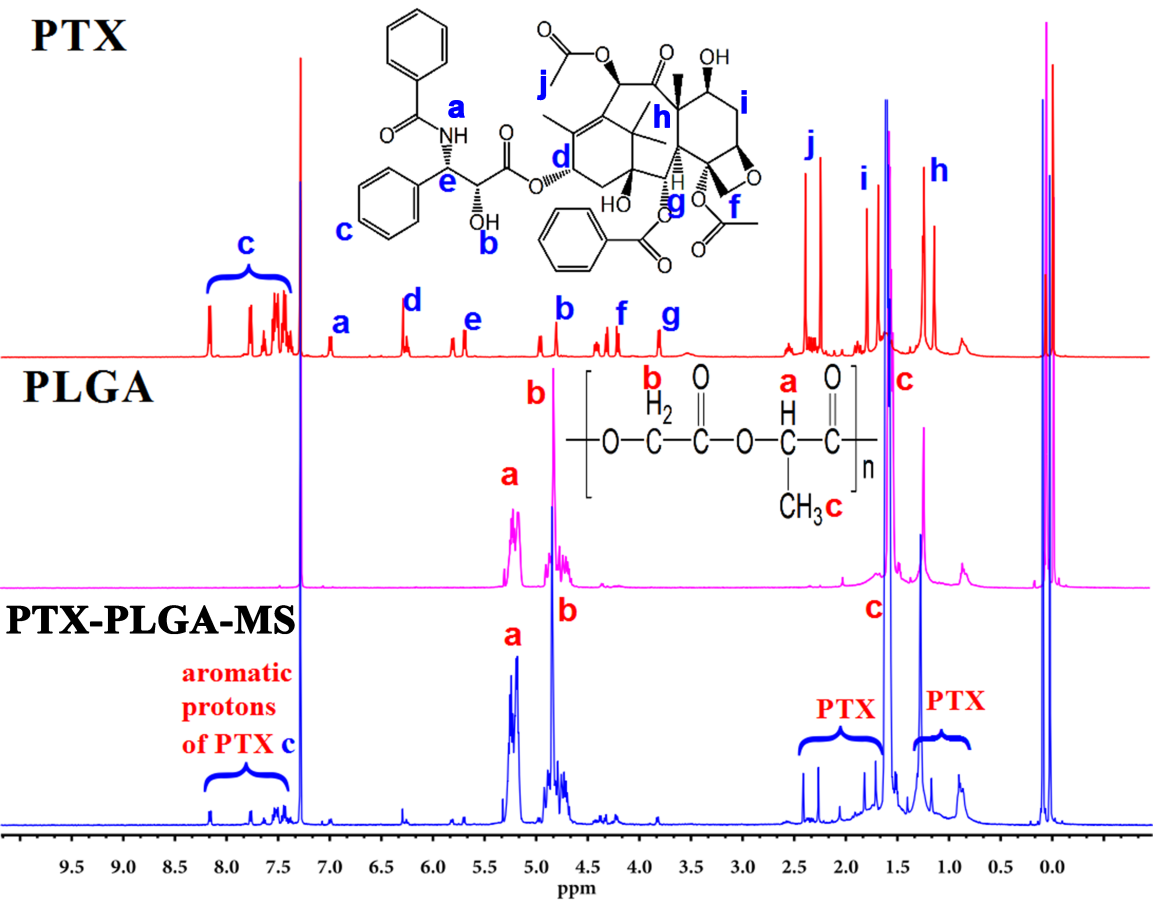


# Figure S1. ^1^H NMR spectra of free PTX, PLGA, and PTX-PLGA-MS in CDCl_3_ with the assignment of the main proton signals.

Table S2. The particle size, drug-loading capacity of smooth and rough PTX-PLGA-MS

| PTX-PLGA-MS types | Drug-loading efficiency (%) | Encapsulation efficiency (%) | Average particle size (μm) |
| --- | --- | --- | --- |
| Smooth MS (A) | 10.13 ± 1.04 | 84.24 ± 2.82 | 67.56 ± 5.73 |
| Smooth MS (B) | 9.79 ± 0.72 | 82.35 ± 2.22 | 62.85 ± 6.06 |
| Smooth MS (C) | 10.85 ± 1.16 | 85.01 ± 1.94 | 70.49 ± 6.46 |
| Rough MS (A) | 15.63 ± 1.38 | 92.82 ± 2.63 | 53.47 ± 2.87 |
| Rough MS (B) | 16.25 ± 0.98 | 93.25 ± 1.95 | 51.35 ± 2.42 |
| Rough MS (C) | 15.96 ± 1.07 | 91.67 ± 2.51 | 49.36 ± 3.17 |

# ***Particle size, drug-loading capacity and in vitro release analysis***


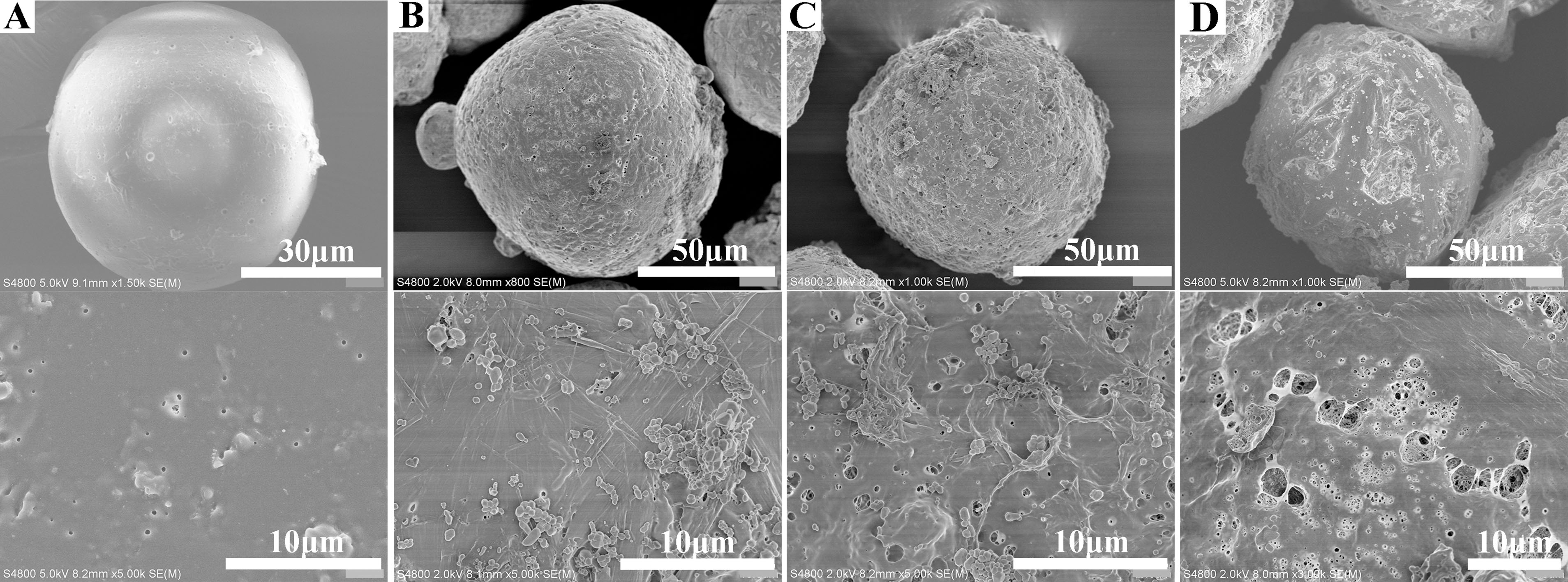


# Figure S2. SEM micrographs of smooth PTX-PLGA-MS after incubation in the release medium for(A) 3 days, (B) 7 days, (C) 14 days, and (D) 21 days.

# **In vitro antitumor activity**


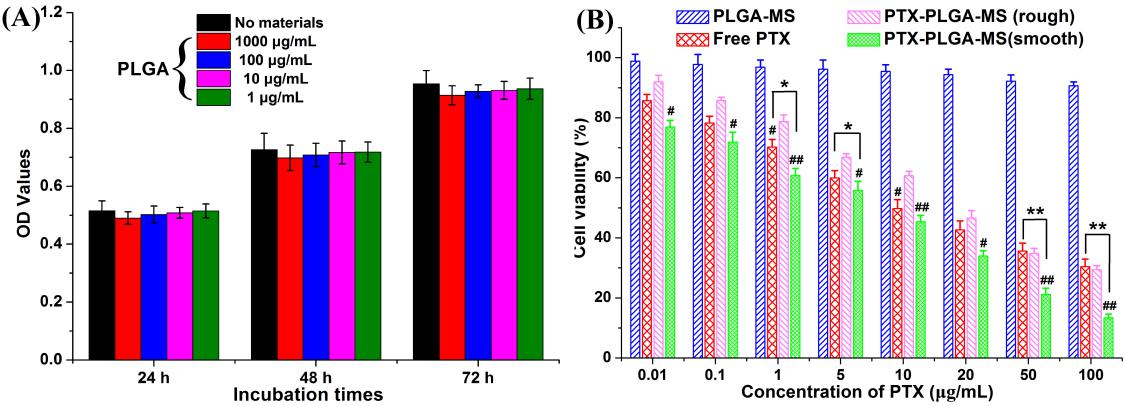


Figure S3. (A): *In vitro* cytotoxicity of PLGA polymer toward U251 cells after incubation with various concentrations. (B): *In vitro* antitumor activities of empty PLGA-MS, free PTX, rough and smooth PTX-PLGA-MS toward U251 cells after incubation for 48 h. # vs. rough PTX-PLGA-MS group, *, # *p* < 0.05, **, ## *p* < 0.02.

**Cell apoptosis and cycle analysis**

Table S3. Cell apoptosis and cell cycle phase distribution of U251 cells with free PTX and rough PTX-PLGA-MS treatments (48 h). The samples concentrations were equivalent to the concentration of PTX.

| Treatment samples  (μg/mL) | Cell apoptosis  (early and late) | Cell cycle phase distribution (%) | | |
| --- | --- | --- | --- | --- |
|  |  | G0/G1 | S | G2/M |
| Control group | 2.91 ± 0.32% | 59.01 ± 3.26 | 31.28 ± 1.24 | 9.71 ± 2.36 |
| Free PTX(25) | 24.47 ± 1.07% | 3.35 ± 1.32 | 3.96 ± 1.69 | 92.69 ± 3.65 |
| Free PTX(5) | 18.31 ± 0.73% | 4.22 ± 0.65 | 5.89 ± 1.57 | 89.89 ± 2.70 |
| Free PTX (1) | 11.37 ± 0.54% | 5.73 ± 0.62 | 7.06 ± 0.75 | 87.20 ± 3.98 |
| Free PTX (0.2) | 5.37 ± 0.37% | 2.88 ± 0.94 | 12.92 ± 0.97 | 84.21 ± 4.22 |
| Control group | 2.69 ± 0.35% | 66.29 ± 1.24 | 5.67 ± 1.26 | 28.04 ± 2.57 |
| PTX-PLGA-MS(25) | 22.03 ± 1.16% | 1.94 ± 0.69 | 6.33 ± 1.32 | 91.73 ± 3.65 |
| PTX-PLGA-MS(5) | 19.38 ± 0.69% | 3.47 ± 0.57 | 6.90 ± 0.65 | 89.63 ± 2.69 |
| PTX-PLGA-MS(1) | 12.44 ± 0.59% | 4.37 ± 0.75 | 6.77 ± 0.62 | 88.86 ± 3.98 |
| PTX-PLGA-MS(0.2) | 7.58 ± 0.46% | 4.21 ± 0.97 | 12.84 ± 0.94 | 82.95 ± 4.21 |

# **Fluoroimmunoassay and western-blot analysis**


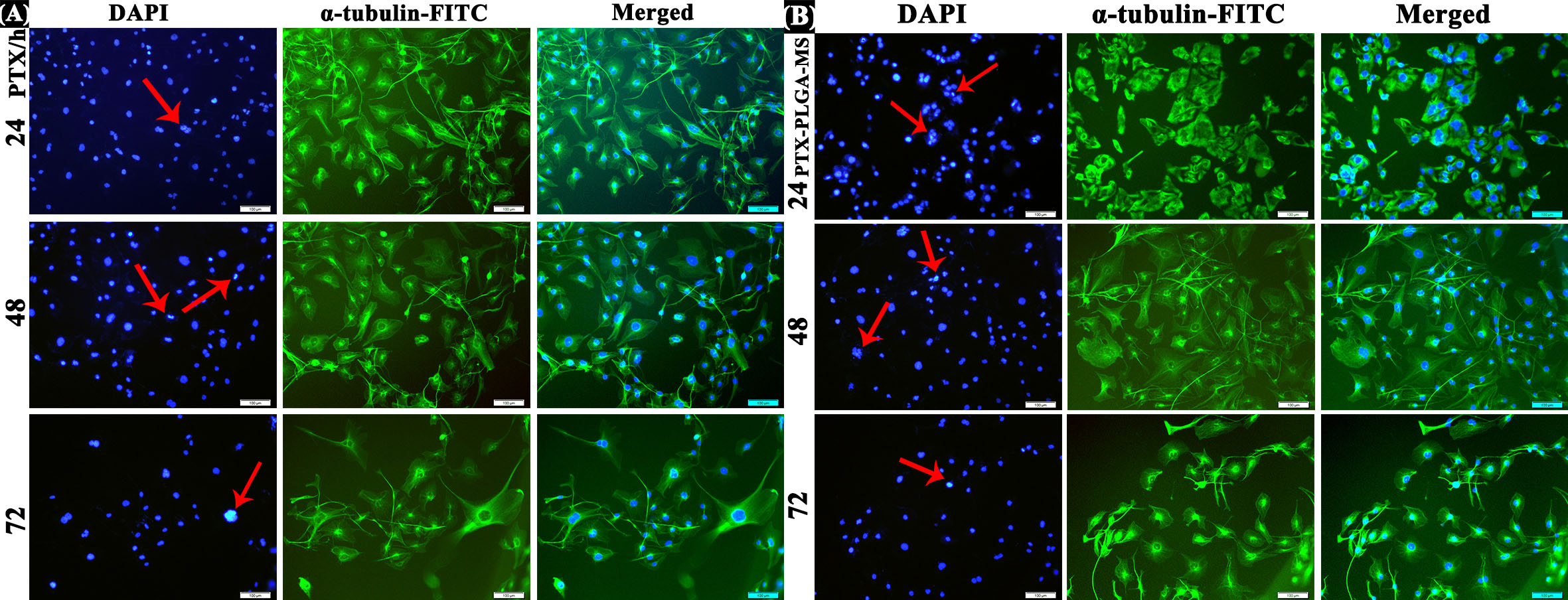


Figure S4. Effects of (A) free PTX and (B) rough PTX-PLGA-MS-induced apoptotic morphological changes on U251 cells. The cells nuclei and cytoplasm were stained by DAPI and α-tubulin-FITC respectively, and visualized under a fluorescence microscope. The red arrows represents the features of apoptotic cells. The scale bars represent 100 μm.


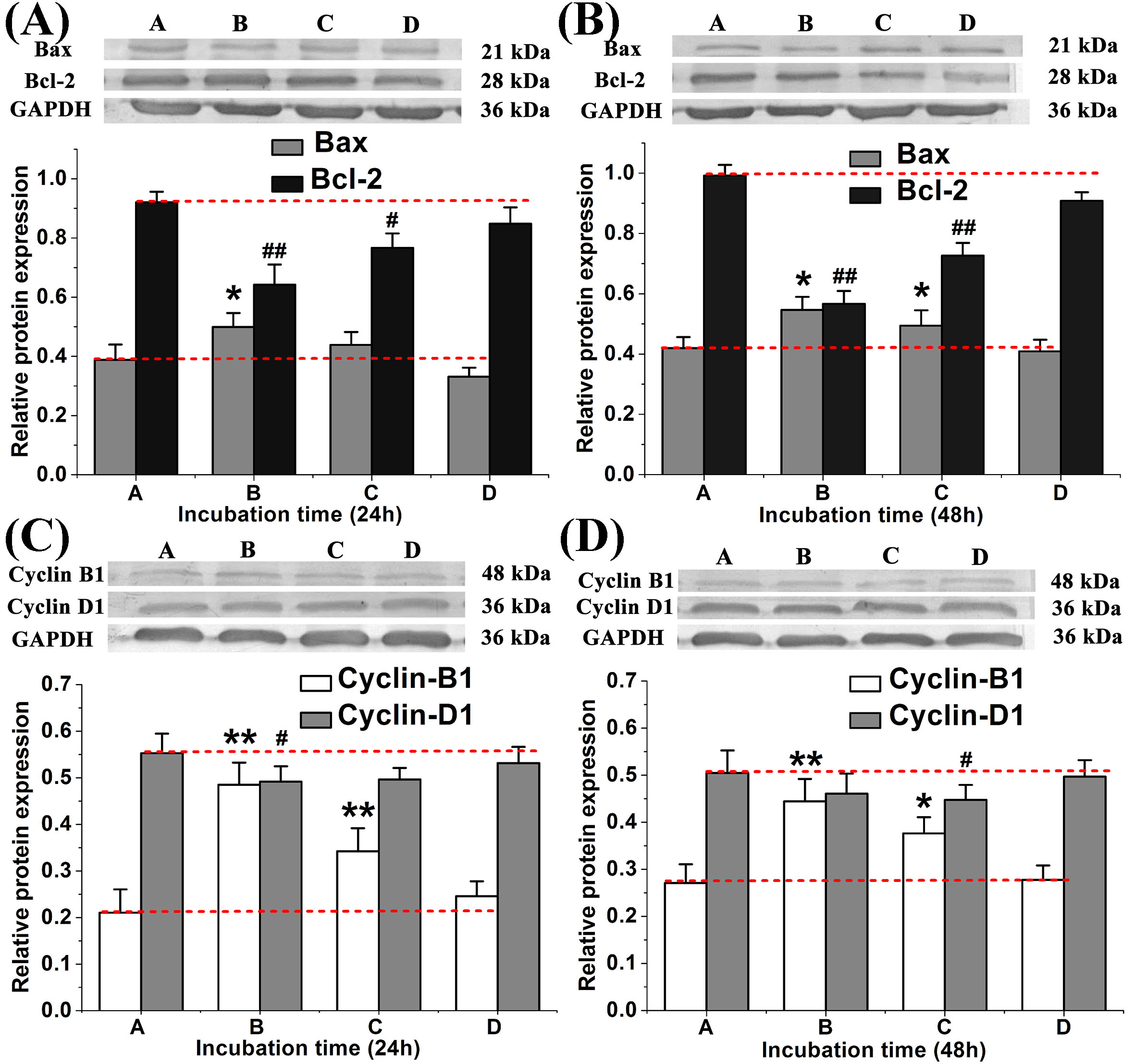


Figure S5. Effects of PTX formulations on the expression of cell apoptosis and cycle-related proteins. A: control group, B: free PTX, C: rough PTX-PLGA-MS, and D: smooth PTX-PLGA-MS. The samples concentration were equivalent to PTX as 5 μg/mL. Western blot analysis and densitometric analysis of Bax/Bcl-2 and Cyclin B1/Cyclin D1 for 24 h (A, C) and 48 h (B, D). Data were normalized to GAPDH. *, # vs. control group, *, # *p* < 0.05, **, ## *p* < 0.02.
